# Revealing the role of laser stimulated radiation rate on laser intensity noise spectrum distribution

**DOI:** 10.1038/s41598-025-88699-6

**Published:** 2025-03-03

**Authors:** Yongrui Guo, Xuesen Xu, Xu Zhou, Nana Zhang

**Affiliations:** 1 College of Optoelectronics Engineering, Chongqing University of Posts and Telecommunications, Chongqing, 400065 China; 2 Post doctoral research workstation of Chongqing Key Laboratory of Optoelectronic Information Sensing and Transmission Technology, Chongqing University of Posts and Telecommunications, Chongqing, 400065 China

**Keywords:** Quantum cascade lasers, Solid-state lasers

## Abstract

In this paper, the influence of the laser stimulated radiation rate on the distribution of the laser intensity noise spectrum is investigated through the analysis of single-frequency lasers with output laser powers ranging from 2 to 140 W and laser stimulated radiation rates varying in order of magnitudes from $10^{10}$ to $10^{11}$ $s^{-1}$. It is found that the magnitude of the stimulated radiation rate can determine the shot noise limit cut-off frequency of the laser, and the specific value of the laser stimulated radiation rate together with the pumping rate and the total photon decay rates, can determine the resonant relation oscillation frequency of the laser. Based on these findings, methods for achieving low intensity noise lasers by obtaining a smaller laser stimulated radiation rate are analyzed and discussed, and a potential approach for realizing low intensity noise high power lasers is proposed.

## Introduction

High-power solid-state continuous-wave single-frequency lasers are widely used in fundamental research including laser precision measurements, ultrafine spectroscopy, cold atomic physics, and so on, owing to their unique features of simultaneously possessing high output power, low intensity noise, and excellent beam quality^1–3^. Especially with the deepening of fundamental scientific research, higher requirements are placed on the intensity noise of these lasers. In the field of weak signal precision measurement, high-power continuous-wave solid-state lasers with extremely low intensity noise can improve the signal-to-noise ratio of laser interferometric measurement devices, thereby improving the measurement accuracy. In cold atomic physics, they can facilitate the preparation of high-barrier magneto-optical traps with high stability. Moreover, in quantum optics, can also be used to create multi-component entangled state light fields for building multichannel quantum network platforms^4^.

To increase the output power of single-frequency solid-state lasers based on a single ring cavity, more incident pumping power needs to be injected. But for a ring cavity with a single laser crystal, more pump power will increase the laser intensity noise caused by amplified spontaneous emission noise. Up to date, the highest output power of a single-frequency laser with a single laser crystal is 50 W, its resonant relation oscillation (RRO) frequency is 938 kHz, and the frequency of the laser spectrum reaching the shot noise limit (SNL) cut-off frequency is above 5.1 MHz^5^. These high-intensity noise characteristics limit its application in basic research. In 2018, a single-frequency laser with an output power of 101 W was achieved by using two independently pumped same laser crystals in a single-ring cavity. The RRO frequency of this laser was about 800 kHz, and the SNL cut-off frequency was above 4.2 MHz, with a total incident pump power of 240 W and an output coupling transmission of 0.37^6^. More recently, a single-frequency laser with an output power of 140 W was realized in a single ring cavity with four independently pumped identical laser crystals^7^. The total incident pump power of this 140 W laser was 370 W, and the output coupling transmission was 0.55, which were both larger than those of the 101 W laser. However, the 140 W laser had lower intensity noise characteristics, such as the RRO frequency of 593 kHz and the SNL cut-off frequency of 2.1 MHz. This indicates that further in-depth analysis based on quantum noise theory is needed to find the key laser intensity noise parameter and understand its influence on the laser intensity noise spectrum distribution.

In this paper, the intensity noise properties of two single-frequency lasers with output powers of 2 and 20 W are compared and analyzed at first. The RRO frequencies and the cut-off frequency for the laser intensity noise spectra reaching SNL are very close, although the output powers and the output coupling transmissions of the two lasers differed by 10 and 5 times. By analyzing the influence of different intensity noise parameters, it is found that compared to laser intensity noise parameters including the pumping rate and the total photon decay rates on laser intensity noise characteristics, the laser stimulated radiation rate has a significant influence on laser intensity noise spectrum distribution. Further comparing the measured intensity noise results of the lasers with different output power levels and different orders of magnitude of laser stimulated radiation rates confirms that the magnitude of the laser stimulated radiation rate can determine the shot noise limit cut-off frequency of the laser, while the specific value of the laser stimulated radiation rate together with the pumping rate and the total photon decay rates can determine the RRO frequency of the laser.

## Laser structures for theoretical simulations

**Fig. 1:**
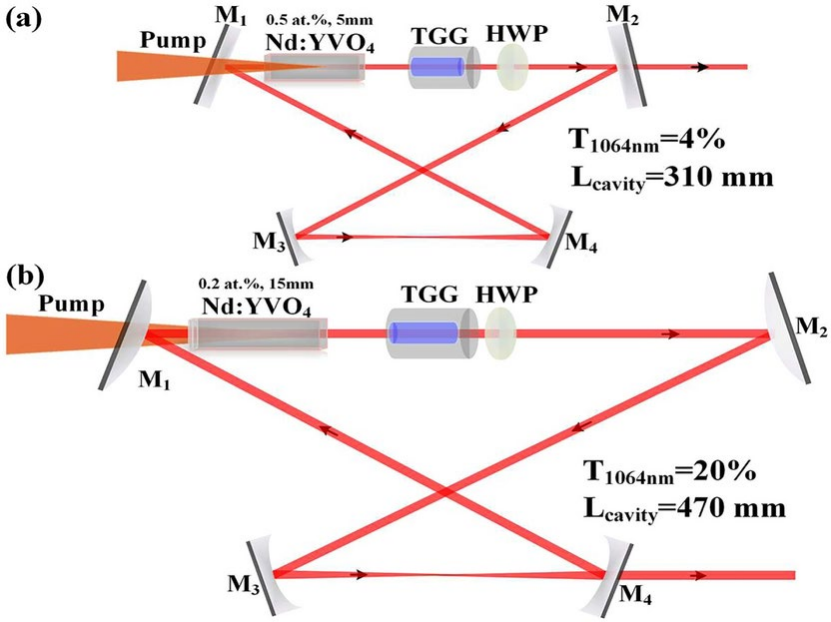
Laser structures of Laser A (**a**) and Laser B (**b**).

The intensity noise properties of two lasers, namely Laser A and Laser B, with output powers of 2 W and 20 W respectively, are compared and analyzed. The laser cavity structures and the laser parameters for Laser A and Laser B are completely referenced to those reported in Ref.^8^ and Ref.^9^, respectively. Both lasers have bow-tie ring cavity, which are depicted in Fig. 1, respectively. The optical cavity lengths of Laser A and Laser B are 310 and 470 mm, respectively. The stable unidirectional operation of Laser A and Laser B is achieved by employing optical diodes composed of a half-wave plate and a terbium gallium garnet (TGG) crystal mounted in hollow cylindrical permanent magnet. The pumping sources of Laser A and Laser B are fiber coupled laser diodes with a center wavelength of 808 nm and maximum output powers of 8 and 50 W, respectively. The corresponding diameters and the numerical aperture of the coupling fibers are 200 $\upmu$m and 0.22, and 400 $\upmu$m and 0.22, respectively. The pump radiations of Laser A and Laser B are focused into the gain media by two sets of lens groups with focal lengths of 30 mm and 50 mm for Laser A, and 30 mm and 80 mm for Laser B. The pump beam waist radii in the central of the gain media for Laser A and Laser B are about 310 and 530 $\upmu$m, respectively. For Laser A, both the input and output coupling mirrors $\hbox {M}_{{1}}$ and $\hbox {M}_{{2}}$ are plane mirrors. The incident and output surfaces of the input mirror $\hbox {M}_{{1}}$ are coated with anti-reflectivity (AR) film at pump laser wavelength of 808 nm ($\hbox {R}_{808\,\hbox {nm}}\,<0.5\%$) and high-reflection (HR) film at lasing wavelength of 1064 nm ($\hbox {R}_{1064\,\hbox {nm}}\,>99.7\%$), respectively. The output coupling mirror $\hbox {M}_{{2}}$ is coated with partial transmission of *T* = 4% at 1064 nm. $\hbox {M}_{{3}}$ and $\hbox {M}_{{4}}$ are both plane-concave mirrors with the curvature radius of 50 mm ($\rho _{2}$ = -50 mm), both are coated with HR film at 1064 nm ($\hbox {R}_{1064\,\hbox {nm}}\,>99.7\%$). The laser crystal is an a-cut Nd:$\hbox {YVO}_{{4}}$ rod, which has a Nd-doped part of 5 mm with the concentration of 0.5 at.%. For Laser B, the input coupling mirror $\hbox {M}_{{1}}$ is a concave-convex lens with the curvature of 1500 mm. The incident and output surfaces of mirror $\hbox {M}_{{1}}$ are coated with AR film at pumping wavelength of 808 nm ($\hbox {R}_{808\,\hbox {nm}}\,<0.5\%$) and HR film at lasing wavelength of 1064 nm ($\hbox {R}_{1064\,\hbox {nm}}\,>99.7\%$), respectively. $\hbox {M}_{{2}}$ is a plane-convex mirror ($\rho _{1}$ = 1500 mm) coated with HR film at 1064 nm ($\hbox {R}_{1064\,\hbox {nm}}\,>99.7\%$). $\hbox {M}_{{3}}$ and $\hbox {M}_{{4}}$ are both plane-concave mirrors with the curvature radius of 100 mm ($\rho _{2}$ = -100 mm). $\hbox {M}_{{3}}$ is coated with HR film at 1064 nm ($\hbox {R}_{1064\,\hbox {nm}}\,>99.7\%$). The output coupling mirror $\hbox {M}_{{4}}$ is coated with partial transmission of *T* = 20% at 1064 nm. The laser crystal is an a-cut composite $\hbox {YVO}_{{4}}$/Nd:$\hbox {YVO}_{{4}}$ rod with an un-doped end cap of 3 mm to reduce the thermal lens effect on the incident end face of the laser crystal and a Nd-doped part of 20 mm with the concentration of 0.8 at.%. For both the lasers, there is a wedge angle of 1.5$^{\circ }$ at the rear end faces of the laser crystals to maintain the stable polarization of the oscillated lasing.

## Simulation results and analysis

For Laser A and Laser B, the beam waist radii in the center of the gain mediums are calculated to be about 200 and 400 $\upmu$m at the maximum incident pump powers of 8 and 50 W upon considering the influence of laser crystal thermal focal length. On this basis, the simulated output powers of $\sim$ 2 and 20 W for Laser A and Laser B upon considering the effect of energy transfer upconversion in lasing formation are close to those reported in Ref.^8^ and Ref.^9^, respectively. The transfer-function-type theory explicitly shows the origin of laser intensity noises by solving the quantum Langevin equation around its steady-state solution with various external quantum-mechanical reservoirs^10^. The intensity noise of the laser can be expressed by Ref.^11^:

1 $$\begin{aligned} \begin{aligned}&V_f=k_1(\omega _f, \gamma _f)V_{vac}+k_2(\omega _f, \gamma _f)V_p+k_3(\omega _f, \gamma _f)V_{spont}+k_4(\omega _f, \gamma _f)V_{dipole}+k_5(\omega _f, \gamma _f)V_{losses}, \end{aligned} \end{aligned}$$

where $k_{1}$, $k_{2}$, $k_{3}$, $k_{4}$, and $k_{5}$ are the coefficients of the vacuum noise $V_{vac}$ caused by the output coupler, $V_{p}$ the noise coming from the pump source, $V_{spont}$ the noise coming from spontaneous-emission, $V_{losses}$ the noise induced by the intra-cavity losses, and $V_{dipole}$ the noise caused by dipole fluctuations, respectively. The $k_{1}$, $k_{2}$, $k_{3}$, $k_{4}$, and $k_{5}$ are the function of RRO frequency $\omega _{f}$ and the damping rate of the oscillation $\gamma _f$. In Eq (1), the pump noise $V_{p}$ is the measured relative intensity noise (RIN), $V_{vac}$=$V_{spont}$=$V_{losses}$=$V_{dipole}$=1 are all at the limit of quantum noise. The RRO frequency $\omega _{f}$ and the damping rate oscillation $\gamma _{f}$ of the laser can be described by:

2 $$\begin{aligned} \omega _{f}=\sqrt{2G\alpha ^2\kappa }, \end{aligned}$$

and

3 $$\begin{aligned} \gamma _{f}f=G\alpha ^2+\Gamma +\gamma _t. \end{aligned}$$

where 2$\kappa$ defines as the total photon decay rate, which can be expressed as: $2\kappa$ = 2$\kappa _{m}$+2$\kappa _{l}$, where 2$\kappa _{m}$ = *tc*/*L* and 2$\kappa _{l}$ = $\delta c/L$ represent the photon decay rates induced by the output losses and the intra-cavity losses, respectively, where *t* is the transmission of the output coupling mirror, $\delta$ is the intra-cavity linear loss, *L* is the optical length of the resonator, and *c* is the velocity of light. The intra-cavity photon number $\alpha ^2$ of the laser can be described by:

4 $$\begin{aligned} \alpha ^2=\frac{j_2(\gamma -\gamma _t)}{2\kappa }-\frac{\gamma _t}{G}, \end{aligned}$$

where $\gamma$ = $\frac{1}{\tau }$ expresses the atomic spontaneous emission rate from lower level to ground state; $\gamma _t$ = $\frac{1}{\tau _{sp}}$ expresses the atomic spontaneous emission rate from upper level to lower level, $\tau _{sp}$ represents the fluorescence lifetime of upper level excitation. where $j_2$ is the probability of ground state particle number distribution, which can be expressed as: $j_{2}$ = $\frac{1-2\kappa /G}{2+\gamma /\Gamma }$, where $\Gamma$ represents the normalized pumping rate of the laser, which can be given by: $\Gamma$ = $\frac{R\eta _{t}\eta _{q}}{\rho }$, where $\eta _{t}$ denotes the optical transmission efficiency of the pumping source, $\eta _{q}$ defines as the quantum efficiency, which is the ratio between the laser frequency $\nu _{l}$ and the pump laser frequency $\nu _{p}$. The pumping rate is described by *R* = $\frac{p_{in}\eta _{a}}{h\nu _{p}V_{mp}}$, where *h* is Planck constant, $\nu _{p}$ is the pump laser frequency, $p_{in}$ is the incident pump power of the laser, $\eta _{a}$=1-$exp(-\beta l)$ expresses the pumping laser absorption efficiency, $\beta$ is the absorption coefficient, $V_{mp}$ represents the mode volume of the pumped laser at the laser crystal, which can be expressed as: $V_{mp}$ = $2\pi \int _{0}^\frac{l}{2}\omega _p^2\sqrt{1+(\frac{\lambda _p z}{\pi \omega _p})^2}dz$, where $\omega _p$ defines as the waist radius of the pumped laser at the center of the laser crystal, $\lambda _p$ represents the wavelength of the pumped laser. In Eq. (2)-Eq. (4), *G* is the laser stimulated radiation rate of the laser, which characterizes the coupling strength between atomic transitions and laser cavity modes, can be expressed by^12,13^:

5 $$\begin{aligned} G=\frac{\sigma \rho l}{n_{r}L/c}, \end{aligned}$$

where *l* represents the ion-doping length of the laser crystal, $\sigma$ is the stimulated emission cross section, $n_{r}$ is the refractive index of the laser crystal. The atomic density is described by: $\rho$ = $\rho _{c}c_{w}$, where $\rho _{c}$ expresses the density of doped ion in 1 at.%, and $c_{w}$ defines as the ion-doping concentration in atomic percentage. To analyze the intensity noise properties of solid-state lasers with different output powers, the intensity noise spectra $V_f$ (1 mW of the detected power) of the lasers should be normalized to the corresponding output powers $p_{out}$ of the laser. According to Ref.^11^, the normalized equation can be newly given by:

6 $$\begin{aligned} V_n=10\lg [1+\frac{V_f-1}{p_{out}}]. \end{aligned}$$

The normalized laser intensity noise spectrum $V_n$ can be simulated by taking the laser parameters into tensity noise simulation program containing Eqs (1)-(6) and running it in software of Mathematica or MATLAB. The relative intensity noise (RIN) of the laser can be obtained by adding the RIN of shot noise for the laser detection power (1 mW) to the normalized laser intensity noise spectrum $V_n$.

**Fig. 2:**
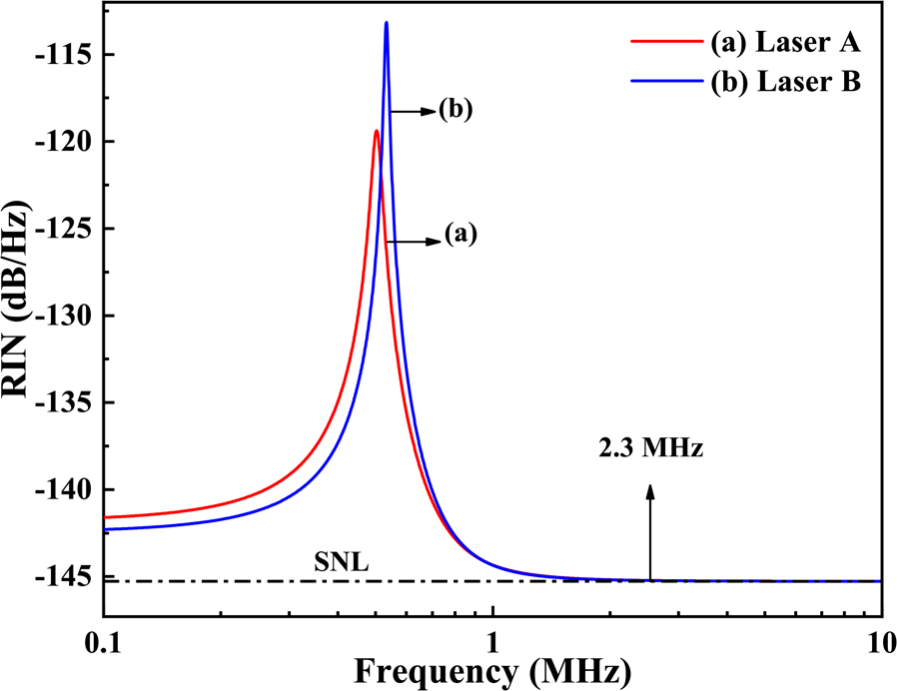
Simulated intensity noise spectra of Laser A and Laser B.

Figure 2 shows the theoretically simulated RIN spectra of Laser A and Laser B, which are obtained by taking the parameters of the Lasers in Table 1 in Eqs (1)-(6). In Fig.2, the RRO frequencies and the frequencies for the RIN spectra researching the SNL cut-off frequency for Laser A and Laser B are 495 and 534 kHz, and above 2.3 MHz, respectively. For the same laser cavity structure and laser parameters of Laser A, it had been reported that the RRO frequency of the laser with the injected pump power of 3.4 W was about 330 kHz^12^. The theoretically simulated RRO frequency of 305 kHz for Laser A at the same incident pump power of 3.4 W is close to the measured value of 330 kHz, indicating the credibility of the simulation results of Laser A. In addition, the reported SNL cut-off frequency of the Nd:$\hbox {YVO}_{{4}}$ laser with the output power of 2 W was above 1.5 MHz after passing through a mode cleaner^8^. Thus, the original SNL cut-off frequency of Laser A should be close to 2.3 MHz. For Laser B, the simulated RRO frequency of 534 kHz is also close to the measured value of 537 kHz for the Nd:$\hbox {YVO}_{{4}}$ laser with similar laser structure and closed laser output power^9,13^. Moreover, the simulated SNL cut-off frequency of 2.3 MHz for Laser B is also close to the measured values of 2.4 MHz reported in Ref^9^. In Fig. 2, the amplitude of the RRO peak for Laser B is higher than that of Laser A, which is the result of stronger dipole fluctuations of Laser B. The dipole fluctuations of a laser come from the dipole coupling between the atoms and the laser resonator, which is influenced by the pumping rate $\Gamma$ and the number of stimulated atoms *N*. Specifically, it has been shown that the RRO frequency of a laser can be completely suppressed and the amplitude of the RRO peak can be reduced by almost 20 dB relative to the SNL when introducing sufficient nonlinear loss into the laser resonator^10,14^. Therefore, after introducing sufficient nonlinear losses into the laser resonators of Laser A and Laser B, the RIN spectra of the lasers are almost similar.

**Table 1:** Parameters for Laser A and Laser B.

Parameter	Laser A	Laser B
$\omega _f$(*kHz*)	495	534
$\gamma _f$($s^{-1}$)	193026	86638
$\Gamma$($s^{-1}$)	265	450
*G*($s^{-1}$)	$3.85\times 10^{10}$	$2.85\times 10^{10}$
$\tau$(*s*)	$1.03\times 10^{-9}$	$1.57\times 10^{-9}$
$2\kappa _t$($s^{-1}$)	$0.73\times 10^{7}$	$1.02\times 10^{7}$
$2\kappa _l$($s^{-1}$)	$1.93\times 10^{7}$	$6.38\times 10^{7}$
$2\kappa$($s^{-1}$)	$2.66\times 10^{7}$	$7.40\times 10^{7}$
*N*(*atoms*)	$9.51\times 10^{16}$	$3.59\times 10^{17}$

The analysis of laser intensity noise spectrum distribution typically focuses on three frequency regions: around the RRO frequency, above, and below the RRO frequency. According to quantum noise theory^11^, the RRO noise of a laser is predominantly determined by several noise sources including the vacuum fluctuation noise introduced by the transmittance of the output coupling mirror, the dipole fluctuations coming from the dipole coupling between the atoms and the laser resonator, and the losses induced by the intra-cavity loss and the output coupling mirror. The intensity noise of the laser below the RRO frequency is influenced by pump noise and spontaneous emission noise. In contrast, the intensity noise above the RRO frequency is mainly governed by the vacuum fluctuation noise. As can be seen from Table 1, the pumping rate, the total photon decay rates resulting from the output loss and the intra-cavity loss, and the normalized atomic number *N* of Laser B are 1.7, 2.8, and 3.8 times those of Laser A, respectively. The laser stimulated radiation rate of Laser A is 1.3 times that of Laser B, yet both are of the same order of magnitude $10^{10}$ $s^{-1}$. It should be noted that according to Eq. (2), the laser stimulated radiation rate *G* is simultaneously related to the number of available atoms *N* and the intra-cavity photon lifetime $\tau$. Specifically, a long intra-cavity photon lifetime implies a small laser stimulated radiation rate, which is beneficial for achieving a low-intensity noise laser^13^. In Table 1, the two lasers have the same order of magnitude for the laser stimulated radiation rate. Consequently, the SNL cut-off frequencies of the two lasers are almost close. Although the slight disparity between the frequency and the peak of RRO of the two lasers is the outcome of the combined influence of the specific value of the laser stimulated radiation rate, the intensity noise parameters of the pumping rate, the total photon decay rates, as well as the normalized atomic number of the laser. The specific value of the stimulated radiation rate for Laser B is slightly smaller than that of Laser A, and the intensity noise parameters including the pumping rate, the total photon decay rates, and the normalized atomic number for Laser B are all larger than those of Laser A.

**Fig. 3:**
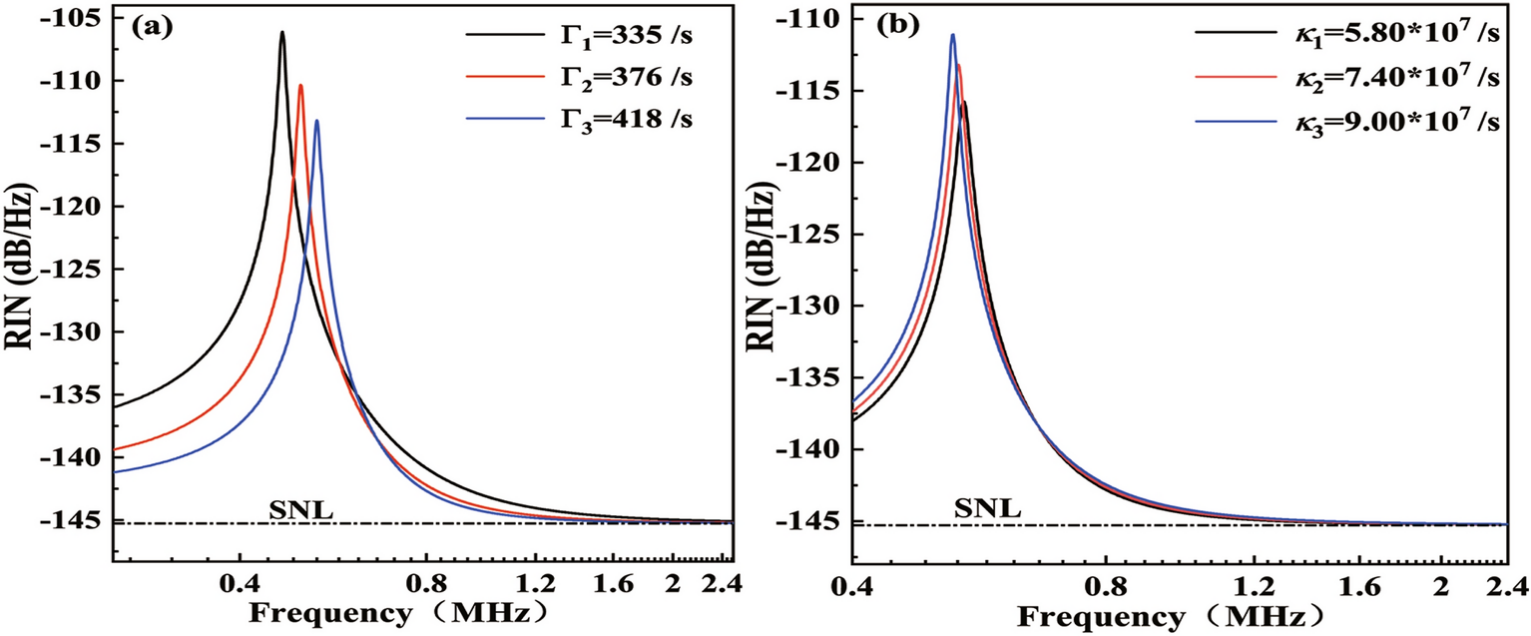
Influence of the pumping rate (**a**) and the total photon decay rates (**b**) on intensity noise of Laser B.

To prove the above points, the impact of other laser intensity noise parameters on the RIN spectrum of the laser is investigated utilizing the control variable method for the RIN of Laser A or Laser B. Compared with Laser A, the pump power and the output coupling mirror transmittance of Laser B can be widely adjusted in the actual design and operation process of Laser B, which is conducive to illustrate the strength of the influence of different laser intensity noise parameters on the RIN spectrum. Therefore, the influence of other laser intensity noise parameters on the RIN spectrum of Laser B is investigated. Figure 3a shows the theoretical simulation results of the influence of different laser pumping rates on the RIN spectrum of the laser. According to Ref.^9^, at incident pump powers of 40, 45, and 50 W, the output powers of Laser B are 4.5, 10.8, and 20.0 W^9^, and the pumping rates of the laser are 355, 376, and 418 $s^{-1}$, respectively. It can be seen that from Fig. 3a, the laser RRO frequency shifts towards the high-frequency range and the amplitude of the RRO frequency decreases with increasing pumping rate. While the cut-off frequency of the laser reaching the SNL hardly changes with the pumping rate. The trend of the simulation results in Fig. 3a is consistent with that of the experimentally measured results in Ref^15^. In addition, the impact of the total photon decay rates on laser intensity noise is theoretically investigated for Laser B with the total photon decay rates of $5.80\times 10^{7}$, $7.40\times 10^{7}$, and $9.00\times 10^{7}s^{-1}$ with respect to the laser output coupling transmissions of 15%, 20%, and 25%, respectively. The simulation results are shown in Fig. 3b. From Fig. 3b, it can be seen that the RRO frequency of Laser B shifts towards the low-frequency range and the amplitudes of the RRO slightly increase with increasing total photon decay rates. However, the SNL cut-off frequency of the laser would not change with the total photon decay rates. The decrease of the laser RRO frequency can be ascribed to the weakened interaction strength between the upper-level inversion population and the oscillating photons resulting from the reduced intra-cavity photon density, whereas the increase of the RRO frequency amplitude can be ascribed to the increased loss noise induced by the output coupling transmissions. The trend of the simulation results in Fig. 3b is also consistent with that of the experimentally measured results in Ref^16^. Therefore, from Fig. 3b, it can be confirmed that the pumping rate and photon decay rates can affect the RRO frequency of the laser but hardly its SNL cut-off frequency.

**Table 2:** Intensity noise parameters of Nd:$\hbox {YVO}_4$ lasers with output power covering from 2 to 140 W.

*P*(*W*)	*G*($s^{-1}$)	*T*	$\omega _f(kHz)$	*SNL*(*MHz*)	$\Gamma$($s^{-1}$)	$\tau$(*ns*)	$2\kappa _t$($s^{-1}$)	$2\kappa _l$($s^{-1}$)	$2\kappa$($s^{-1}$)
2	$3.85\times 10^{10}$	0.04	495	2.4	265	1.00	$2.00\times 10^{7}$	$7.50\times 10^{6}$	$2.75\times 10^{7}$
20^9^	$2.85\times 10^{10}$	0.20	535	2.4	450	1.57	$6.38\times 10^{7}$	$1.02\times 10^{7}$	$7.40\times 10^{7}$
140^7^	$9.00\times 10^{10}$	0.55	593	2.1	184	5.67	$4.85\times 10^{7}$	$4.41\times 10^{6}$	$5.29\times 10^{7}$
30^9^	$1.75\times 10^{11}$	0.20	809	4.2	156	1.50	$6.66\times 10^{7}$	$1.50\times 10^{7}$	$8.16\times 10^{7}$
50^5^	$3.40\times 10^{11}$	0.25	938	5.1	115	1.50	$9.32\times 10^{7}$	$1.67\times 10^{7}$	$9.99\times 10^{7}$
100^6^	$1.14\times 10^{11}$	0.37	802	4.3	230	3.16	$3.16\times 10^{7}$	$7.90\times 10^{6}$	$3.95\times 10^{7}$

To further confirm the role of laser stimulated radiation rate on laser intensity noise spectrum distribution, the intensity noise characteristics of the Nd:$\hbox {YVO}_{{4}}$ lasers with output laser powers ranging from 2 to 140 W and laser stimulated radiation rates with magnitudes from $10^{10}$ to $10^{11}s^{-1}$ are analyzed. The output coupling transmissions of the Nd:$\hbox {YVO}_{{4}}$ lasers with output powers of 30^9^, 50^5^, 101^6^, and 140 W^7^ are the actual values of the lasers, and the RRO frequency and the SNL cut-off frequency of the lasers are all the experimentally measured values, which are shown in Table 2. The intensity noise parameters in Table 2, including pumping rate $\Gamma$, the intra-cavity photons lifetime $\tau$, photon decay rates of $2\kappa _t$ and $2\kappa _l$ in Table 2 are all calculated based on the actual parameters of the lasers^17^. For the Nd:$\hbox {YVO}_{{4}}$ lasers with output powers of 30, 50, 100, and 140 W, the optical cavity lengths of the lasers are 450, 450, 948, and 1700 mm, the measured RRO frequencies and the SNL cut-off frequencies of the lasers are 809 kHz and 4.2 MHz, 938 kHz and 5.1 MHz, 802 kHz and 4.3 MHz, and 593 kHz and 2.1 MHz, and the calculated laser stimulated emission rate of the lasers are $1.75\times 10^{11}$, $3.40\times 10^{11}$, $1.14\times 10^{11}$ and $9.00\times 10^{10}$ $s^{-1}$, respectively. From Table 2, it can be seen that for the Nd:$\hbox {YVO}_{{4}}$ lasers with output powers of 2, 20, and 140 W, the laser stimulated radiation rate for the lasers have the same magnitude of $10^{10}$ $s^{-1}$, and both the RRO frequencies and SNL cut-off frequencies of the lasers are very close. The same phenomenon can be also observed from the Nd:$\hbox {YVO}_{{4}}$ lasers with output powers of 30, 50, and 101 W, where the laser stimulated radiation rates for the lasers are with the same magnitude of $10^{11}$ $s^{-1}$. In addition, both the RRO frequency and SNL cut-off frequency of the lasers with the laser stimulated radiation rate magnitude of $10^{11}$ $s^{-1}$ are larger than those of the lasers with the laser stimulated radiation rate magnitude of $10^{10}$ $s^{-1}$. Therefore, it can be deduced that the magnitude of the laser stimulated radiation rate can determine the frequency regions of both the RRO frequency and SNL cut-off frequency of the laser. When comparing the RRO frequencies of the Nd:$\hbox {YVO}_{{4}}$ lasers with the same magnitude of the laser stimulated radiation rates in Table 2, it can also be concluded that the specific value of laser stimulated radiation rate together with the pumping rate, the total photon decay rates can determine the specific value of laser RRO frequency. In addition, by comparing the intensity noise parameters of Nd:$\hbox {YVO}_{{4}}$ lasers with output powers of 101 and 140 W, it can be found that the intra-cavity photons lifetime also has a significant impact on the laser intensity noise characteristics, including the RRO frequency and the SNL cut-off frequency of the laser. This is because a longer intra-cavity photons lifetime can enhance the laser cavity noise filtering capability^18^.

## Discussion

**Fig. 4:**
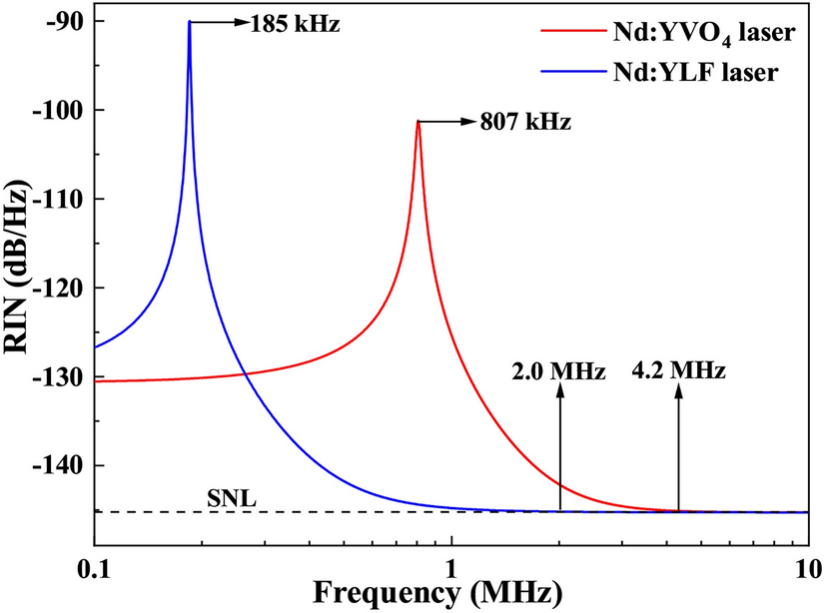
Intensity noise spectra of Nd:$\hbox {YVO}_{{4}}$ and Nd:YLF lasers.

Based on the above analysis, it can also be concluded that reducing the laser stimulated radiation rate of the laser is beneficial for the realization of low-intensity noise lasers. According to Eq. (5), choosing a laser crystal with a smaller stimulated emission cross section is the most direct way to achieve a lower laser stimulated radiation rate of the laser. However, the small stimulated emission cross-section of the laser crystal will severely limit the output power of the laser^19^. Increasing the optical cavity length of the laser can effectively reduce the laser stimulated radiation rate, thereby significantly suppressing the laser intensity noise over a wide frequency range^13^. Nevertheless, in the design of the laser cavity, how to ensure efficient mode matching between the pumping laser and the cavity mode in the laser crystal for the long laser resonator cavity must be carefully considered. The imaging system should be introduced into the long laser cavity for adjusting the oscillating lasing beam waist at the laser crystal. Additionally, changing the doping length or doping concentration of the laser crystal to decrease the laser stimulated radiation rate of the laser must be carefully considered, and the appropriate pumping scheme should be employed to ensure high optical-to-optical conversion efficiency^9^. Selecting laser crystals with a smaller stimulated emission cross section but stronger heat dissipation capacity to handle high incident pump power is a potential way to achieve high-power lasers with low-intensity noise. For the generation of lasing wavelength at $\sim$ 1 $\upmu$m, the stimulated emission cross-section of $1.2\times 10^{-19}$ $\hbox {cm}^2$ for Nd:$\hbox {LiYF}_{{4}}$ (Nd:YLF) crystal at 1053 nm is 20.8 times smaller than that of $25.0\times 10^{-19}$ $\hbox {cm}^2$ for Nd:$\hbox {YVO}_{{4}}$ crystal at 1064 nm, and the thermal conductivity of 6.3 W/m/K for Nd:YLF crystal is larger than that of 5.23 W/m/K for Nd:$\hbox {YVO}_{{4}}$ crystal^20^. Presently, the reported incident pump power for the bulk Nd:YLF crystal can exceed 100 W, and the output power of the Nd:YLF laser has reached more than 30 W by employing the direct pumping scheme^21–23^. Figure 4 depicts the simulated RIN spectra of Nd:$\hbox {YVO}_{{4}}$ and Nd:YLF lasers with output powers of $\sim$ 30 W. The RIN spectra of the lasers are obtained by substituting the laser parameters in Table 3 into Eq. (1). From Fig. 4, it can be clearly seen that for the Nd:YLF laser, the RRO frequency is 185 kHz and the SNL cut-off frequency is 2.0 MHz, which are smaller than those of 807 kHz and 4.2 MHz for the Nd:$\hbox {YVO}_{{4}}$ laser, showing the advantage of the Nd:YLF crystal in achieving low intensity noise high-power lasers. In addition, after introducing sufficient non-linear losses into the lasers cavity, the RRO frequencies of the lasers can be completely suppressed^24^.

**Table 3:** Parameters for intensity noise simulation of Nd:$\hbox {YVO}_{{4}}$ and Nd:YLF lasers.

Parameters	$c_w\,(at.\%)$	$l\,(mm)$	$P_{in}\,(W)$	*t*	$L\,(mm)$	$\omega _{p}\,(\mu m)$	$\eta _a$	$V_{p}\,(dB)$	$\rho _{c}\,(at/cm^{3})$	$\tau _{f}\,(\mu s)$	$G\,(s^{-1})$
$Nd:YVO_{4}$	0.8	20	80	0.20	450	510	0.89	12.8	$1.26\times 10^{20}$	100	$1.75\times 10^{11}$
*Nd* : *YLF*	1.0	30	90	0.20	450	510	0.80	12.8	$1.40\times 10^{20}$	485	$2.30\times 10^{10}$

## Conclusion

This paper explores the significance of the laser stimulated radiation rate in laser intensity noise spectrum distribution. The intensity noise properties of two single-frequency lasers are compared: one with an output power of 2 W and an output coupling transmission of 0.04, and the other having an output power of 20 W and an output coupling transmission of 0.20. Furthermore, an in-depth analysis is conducted on how intensity noise parameters affect the laser intensity noise characteristics. It is found that the magnitude of the laser stimulated radiation rate plays a crucial role in determining the SNL cut-off frequency of the laser. Additionally, the specific value of the laser stimulated radiation rate, when combined with the pumping rate and the total photon decay rates, is instrumental in determining the RRO frequency of the laser. To further confirm the impact of the laser stimulated radiation rate on laser intensity noise, a comparative analysis is carried out on the experimentally measured intensity noise characteristics of lasers. These lasers have output powers ranging from watt level to hundred-watt level and exhibit different magnitudes of the laser stimulated radiation rate. On this basis, this paper analyzes and deliberates on methods to achieve low intensity noise lasers by reducing the laser stimulated radiation rate. In addition, a potential approach to realize low intensity noise high power lasers by selecting the laser crystal with a smaller stimulated emission cross-section but stronger heat dissipation capability is proposed. We believe that this paper can provide a good reference for understanding the role of laser stimulated radiation rate on laser intensity noise spectrum distribution, as well as offer a useful reference for achieving low-intensity noise high-power lasers.

## Data Availability

The datasets used and/or analyzed during the current study are available from the corresponding author upon reasonable request.
